# Tumor enlargement in adrenal incidentaloma is related to glaucoma: a new prognostic feature?

**DOI:** 10.1007/s40618-023-02154-9

**Published:** 2023-07-19

**Authors:** M. Caputo, T. Daffara, A. Ferrero, M. Romanisio, E. Monti, C. Mele, M. Zavattaro, S. Tricca, A. Siani, A. Clemente, C. Palumbo, S. De Cillà, A. Carriero, A. Volpe, P. Marzullo, G. Aimaretti, F. Prodam

**Affiliations:** 1 Endocrinology, Department of Translational Medicine, Università del Piemonte Orientale, via Solaroli 17, 28100 Novara, Italy; 2 Department of Health Sciences, Università del Piemonte Orientale, via Solaroli 17, 28100 Novara, Italy; 3 Department of Diagnostic and Interventional Radiology, Department of Translational Medicine, Università del Piemonte Orientale, Novara, Italy; 4 Ophthalmology Unit, Department of Health Sciences, Università del Piemonte Orientale, Novara, Italy; 5 Division of Urology, Department of Translational Medicine, Università del Piemonte Orientale, Novara, Italy

**Keywords:** Adrenal, Incidentaloma, Glaucoma, Enlargement

## Abstract

**Purpose:**

The uncertainty on the management of small adrenal incidentalomas (AIs) still represents a challenge in real clinical practice. Considering the lack of knowledge on risk factors implicated in tumour enlargement, the aim of this study was to identify risk factors for morphological changes during follow-up of adrenal incidentalomas (AIs).

**Methods:**

We retrospectively evaluated demographic, clinical, radiological and biochemical parameters of 153 AIs (2007–2021). Patients with histological diagnosis of metastases or pheochromocytoma were excluded. To detect risk factors for tumor enlargement, diseases associated with AIs were included if their prevalence was higher than 2%. Patients were divided into two groups (A: radiological stability; B: tumor enlargement defined as > 5 mm/year in the main diameter).

**Results:**

Group A: 89.5% and group B: 10.5%, mean follow-up 38.6 ± 6.9 months (range 6–240). Tumor enlargement when occurred was within 36 months of follow-up. In group B high body weight (*p* < 0.03), dehydroepiandrosterone sulfate (DHEAS) (*p* < 0.05) and direct renin concentration (DRC) (*p* < 0.04) were higher than group A, while aldosterone levels were lower; moreover, considering comorbidities, glaucoma and dysglycemia (*p* < 0.01 for both) had higher prevalence in group B. Glaucoma and dysglycemia were independent predictors of enlargement. Patients affected by glaucoma, atrial fibrillation, dysglycemia had a lower dimensional change-free survival than non-affected.

**Conclusions:**

Glaucoma might be a novel risk factor for AI enlargement. If subtle undetectable cortisol hypersecretion has a role is a topic for further research.

## Introduction

Adrenal incidentaloma (AI) is defined as an adrenal lesion detected on imaging not performed for suspected adrenal disease. The prevalence of AI has increased from 0.35 to 1.9% in the radiological series of the 90 s [1, 2], to 4.2–7.3% of nowadays, and rises up to 10% among elderly population [2–5] considering the increase of lifespan and the improvement in radiological techniques.

Non-functioning adrenocortical adenomas currently represent the majority of AIs; however, hormone-producing adenomas (i.e., cortisol or aldosterone-producing adenoma), pheochromocytoma, or malignancies (i.e., adrenocortical carcinoma and metastasis) can be found in a minority of cases. Moreover, up to 25–30% of patients with AI shows autonomous cortisol secretion (ACS) [2, 3, 6, 7], defined as a low-grade ACTH independent cortisol excess that could be associated with comorbidities as arterial hypertension, type 2 diabetes mellitus (T2DM), obesity, dyslipidemia, and osteoporosis [2, 8–11].

Management of AI is currently debated. The need of a multidisciplinary evaluation and surgical approach in case of significant hormonal excess and/or radiological suspicious of malignancy is well established [2, 3]. On the other hand, the optimal approach (both clinical and radiological) for surveillance of benign-appearing AI to detect malignant transformation and surgical decision making are still controversial [3, 12–15, 18].

To date, according to the European Society of Endocrinology (ESE)/European Network for the Study of Adrenal Tumors (ENSAT) guidelines [3] and Korean guidelines [14], no further imaging is required for adrenal masses smaller than 4 cm with benign features. However, other recommendations indicated the need of periodic radiological follow-up (i.e., 3–6 up to 12–24 months, CT scan, regardless from mass size at diagnosis) [12, 13, 15–18].

The lack of consensus on AIs management has clinical impact and cost-effectiveness implications. Chomsky-Higgins et al. [19] developed a model evaluating different surveillance strategies for management of small (< 4 cm), benign-appearing AI. According to this paper, no or single surveillance showed favorable outcomes in terms of cost-effectiveness, while closer surveillance was less effective and costlier due to false positives cases, leading to unnecessary surgery and exposure to radiation. Furthermore, an age-dependent management could be considered, since the single surveillance strategy was cost-effective in younger patients (< 60 years), whereas in older patients (> 60 years) the no-surveillance strategy was more effective [19].

Furthermore, there are no evidence-based cut-offs regarding the extent of tumor increase suggesting malignancy and surgical resection. Surgical resection is suggested with a 5 mm or 20% growth in size in 6–12 months [3], or 0.8–1 cm [13] or 0.5–1 cm [14] according to different guidelines.

Recently, a systematic review and meta-analysis confirmed that most of non-functioning adrenal adenomas and adenomas causing mild ACS did not change significantly and 2.5% only grew by 10 mm or more over a mean follow-up of 41.5 months without becoming malignant [20].

Considering the lack of knowledge on risk factors implicated in tumour enlargement, the aim of our study was to identify potential predictors and risk factors of morphological changes by retrospectively analyzing clinical, radiological and pathological features of AIs referred to our center.

## Patients and methods

### Patients

An observational, retrospective, single-center study was performed. Data from 177 adult outpatients affected by AIs and referring to “Maggiore della Carità” University Hospital between the 01st of January 2007 and 31st of March 2021 were consecutively collected. We excluded from the cohort patients with histological diagnosis of metastases or pheochromocytoma.

The protocol for evaluation of all patients was the routine clinical examination of subjects with AIs, including hormonal and periodical clinical and radiological assessments. The study was conducted in accordance with the Declaration of Helsinki, approved by the Local Ethical Committee (AOU “Maggiore della Carità” Novara). Informed consent was obtained from each patient.

Clinical-anthropometric and hormonal characteristics of all subjects were evaluated through the review of endocrine clinical records.

The radiological characteristics of AIs were analyzed by CT or MRI investigation, accordingly to the first methodology performed in each patient at diagnosis and during follow-up.

The follow-up time was calculated in months for each subject, considered as the interval between the first and the last morphological investigation (minimum 1 year).

According to the size of AIs during follow-up, patients were divided into 2 groups: the group A included patients with radiological stability of AI, while group B included patients with increase in tumor size (defined as > 5 mm/year in the main diameter).

For each patient, the following data were collected:

- Demographic characteristics (sex, age at diagnosis);
- Clinical characteristics and features known to be associated with adrenal hormone hypersecretion (body weight, height, body mass index (BMI), smoking habits, comorbidities as T2DM, arterial hypertension, osteoporosis, dyslipidemia, atrial fibrillation)
- Any other reported diseases;
- Concomitant medications;
- Radiological parameters: localization (right, left, bilateral), major diameter; for unenhanced CT scan, the attenuation of signal density expressed in HU was used to categorize lesions (≤ 10 HU vs > 10 HU); for MRI images, the intensity in T1 and T2 and the loss of intensity at chemical shift were evaluated;
- Biochemical parameters: plasma sodium and potassium, plasma cortisol after suppression test with dexamethasone 1 mg (DST), urinary free cortisol (UFC) and cortisone; ACTH (in patients with ACS); direct renin activity (DRC) and aldosterone (in hypertensive or hypokalemic subjects) and aldosterone renin ratio (ARR); DHEAS, androstenedione, testosterone/oestrogen and 17-OH progesterone (17OHP) (for patients suspected for adrenal cortical carcinoma and/or hyperandrogenism or bilateral adrenal masses).

In patients undergoing surgery, the surgical approach (laparoscopy, open, robotic) and histopathological data were recorded.

### Methods

#### Radiological evaluation

All the radiological investigations were carried out at the Institute of Diagnostic Radiology of the “Maggiore della Carità” University Hospital of Novara and all the images were re-evaluated by two independent specialists in Radiodiagnostics, with specific expertise in adrenal pathology. CT scans were performed using a Philips 128-layer Ingenuity CT Elite scanner by 2 mm thickness for each scan, incrementable by 1 mm (128 kV, dose right index 17, mAs 100 and 500). The scan time was 3.3 s with pitch 1.49 and rotation time 0.4 s.

MRI mass acquisitions were performed using a Philips Ingenia 1.5 T MRI using Dixon all black T1 weighted, Dixon in and out-of-phase, TSE T2, T2-Spir and DWI sequences with adc map calculation.

The radiological evaluations at follow-up were performed with the same method used at baseline.

#### Biochemical and hormonal evaluations

All the hormonal evaluations were performed at the Laboratory of our Hospital (currently representing one out of two regional hubs).

The serum levels of aldosterone, renin, ACTH, cortisol, DHEA-S and androstenedione were measured by the chemiluminescence technique using the LIAISON^®^ kits.

For ARR calculation, the limit of 3.7 was considered (aldosterone expressed in ng/dl and DRC in mU/L). Aldosterone and DRC analysis were performed after therapy wash-out, if required to correctly identify the presence of primary aldosteronism.

Serum levels of 17-OHP were measured by ELISA technique using the DRG^®^ kit.

Cortisol and urinary cortisone were measured by high performance liquid chromatography-mass spectrometry (HPLC–MS) using the kit produced by B.S.N. srl; the normal range for adults is 3.5–45 µg / day for cortisol and 17–129 µg/day for cortisone, respectively.

To assess 1 mg DST, patients were educated to assume 1 mg of dexamethasone orally between 11.00 pm and 00.00 am the day before the test; cortisol sample was performed between 8.00 and 9.00 am.

A cortisol value < 1.8 μg/dl excluded ACS, while values > 5 μg/dl confirmed it. A cortisol after suppression test between 1.8 and 5 μg/dl identified possible ACS [3].

The presence of type 2 diabetes mellitus (T2DM), impaired fasting glucose (IFG) or impaired glucose tolerance (IGT) was assessed according to current guidelines [21] based on plasma glucose criteria, either the fasting plasma glucose value or the 2 h plasma glucose value during a 75 g oral glucose tolerance test (OGTT), or glycosylated hemoglobin (A1C) criteria. In particular, T2DM was defined for fasting plasma glucose ≥ 126 mg/dL (7.0 mmol/L) or 2 h plasma glucose ≥ 200 mg/dL (11.1 mmol/L) during OGTT OR A1C ≥ 6.5% (48 mmol/mol) or in a patient with classic symptoms of hyperglycemia or hyperglycemic crisis, a random plasma glucose ≥ 200 mg/dL (11.1 mmol/L). In the absence of unequivocal hyperglycemia, diagnosis requires two abnormal tests. IFG was defined as fasting plasma glucose 100 mg/dL (5.6 mmol/L) to 125 mg/dL (6.9 mmol/L); IGT was defined as 2 h plasma glucose during 75 g OGTT 140 mg/dL (7.8 mmol/L) to 199 mg/dL (11.0 mmol/L).

Dyslipidemia was diagnosed and treated according to cardiovascular risk [22].

#### Histological evaluations

All histological reports were evaluated by two different pathologists. The following data were reported: macroscopic diameter, presence or absence of necrosis, number of mitoses, lymph vascular invasion, surgical margins. Capsule invasion, architectural growth pattern, eosinophilic cells, presence, or absence of nucleolar atypia (Fuhrman grade) were also recorded to calculate Weiss Score.

Immunohistochemically evaluation was performed in case of pheochromocytoma, suspected metastatic lesions or adrenocortical carcinoma.

### Statistical analysis

The results are expressed as mean ± SD, absolute values, or percentage. To detect new risk factors, further diseases associated to AIs were included if their prevalence was higher than 2%. Chronic obstructive pulmonary disease (COPD), neoplasms, and glaucoma answered to this criterion and were included in the following analyses.

Difference between groups and continuous variables was analyzed by the Student’s *t* test, while for the dichotomous variables the distribution was evaluated using the Chi-square test.

The association between continuous factors was evaluated by the correlation analysis (Pearson’s *r*). Multivariate Cox-regression was performed, using Hazard ratio (HR) with 95% confidence intervals (CI). Dimensional changes over time were used as dependent variables. Age, sex, weight, arterial hypertension, dyslipidemia, osteoporosis, hyperglycemia, glaucoma, atrial fibrillation, COPD, statin therapy, cortisol at the DST (yes/no), aldosterone, renin, ARR, HU, surgical approach were used as factors or covariates.

The analysis of the historical series was used to evaluate the differences in the detection time of the dimensional increases of the lesions. The Kaplan–Meier curves with the log rank test were used to evaluate the factors associated with the dimensional increase over the follow-up time.

*P* < 0.05 were considered significant. Analyses were performed using SPSS 26.0 for Windows (SPSS, IBM, USA).

## Results

Excluding patients with histological diagnosis of adrenal metastases or pheochromocytoma, 153 AIs were definitively included in the study.

Most of the patients were females (*N* = 89, 58.4%, F/M 1.3/1). Age at diagnosis was 59.8 ± 11.4 years (range 27–82 years).

Mean follow-up was 43.8 ± 45.7 months. Unchanged over time AIs were the 89.5% (*N* = 137) (group A, follow-up 40.5 ± 43.4 months), while 10.5% (*N* = 16) showed tumor enlargement (group B, follow-up 84.7 ± 55.6 months, *p* = 0.005). Following paragraphs describe phenotypical characteristics according to groups and as a whole. Four patients were lost at follow-up (after 8–108 months) and were included considering them stable until the last observation.

### Clinical characteristics

Clinical characteristics of the enrolled patients are reported in Table 1.

**Table 1:** Clinical characteristics of patients affected by AI (whole cohort and subgroups)

Variables	Total *N* (%)	Unchanged (A) *N* (%)	Changed (B) *N* (%)	*p* value
	153 (100%)	137 (89.5%)	16 (10.5%)	
Sex				
F	89 (58.2%)	80 (58.4%)	9 (56.2%)	ns
M	64 (41.8%)	57 (41.6%)	7 (43.8%)	
Age (years)	59.8 ± 11.4	59.9 ± 11.3	58.6 ± 12.2	ns
Weight (kg)	77.1 ± 17.5	75.2 ± 16.8	87.3 ± 18.2	*p* < 0.03
BMI (Kg/m^2^)	31.8 ± 13.4	31.1 ± 4.9	37.5 ± 13.4	ns
Smoking history				
Yes	34 (22.3%)	28 (20.4%)	6 (37.5%)	ns
No	25 (16.3%)	20 (14.6%)	5 (31.25%)	
Unknown	94 (61.4%)	89 (65.0%)	5 (31.25%)	
Arterial hypertension				
Yes	102 (66.6%)	89 (65.0%)	13 (81.25%)	ns
No	39 (25.6%)	37 (27.0%)	2 (12.5%)	
Unknown	12 (7.8%)	11 (8.0%)	1 (6.25%)	
T2DM				
Yes	30 (19.6%)	24 (17.5%)	6 (37.5%)	*p* < 0.01
IGT	6 (4.0%)	6 (3.6%)	0 (0%)	
No	103 (67.3%)	96 (70.1%)	8 (50.0%)	
Unknown	14 (9.1%)	12 (8.8%)	2 (12.5%)	
Osteoporosis				
Yes	19 (12.5%)	19 (13.9%)	0 (0.0%)	ns
No	122 (79.7%)	107 (78.1%)	15 (93.7%)	
Unknown	12 (7.8%)	11 (8.0%)	1 (6.3%)	
Dyslipidemia				
Yes	49 (32.0%)	43 (31.4%)	6 (37.5%)	ns
No	92 (60.2%)	83 (60.6%)	9 (56.25%)	
Unknown	12 (7.8%)	11 (8.0%)	1 (6.25%)	
Statin use				
Yes	40 (26.1%)	36 (26.3%)	4 (25.0%)	ns
No	92 (60.1%)	81 (59.1%)	11 (68.75%)	
Unknown	21 (13.8%)	20 (14.6%)	1 (6.25%)	
Type of statin				
Rosuvastatin	5 (3.3%)	4 (11.1%)	1 (25.0%)	ns
Simvastatin	14 (9.1%)	13 (36.1%)	1 (25.0%)	
Atorvastatin	21 (13.7%)	19 (52.8%)	2 (50.0%)	
Atrial fibrillation				
YES	10 (6.6%)	7 (5.1%)	3 (18.75%)	ns
NO	131 (85.6%)	119 (86.9%)	12 (75.0%)	
Unknown	12 (7.8%)	11 (8.0%)	1 (6.25%)	
Glaucoma				
Yes	5 (3.3%)	2 (1.5%)	3 (18.8%)	*p* < 0.01
No	136 (88.9%)	124 (90.5%)	12 (75.0%)	
Unknown	12 (7.8%)	11 (8.0%)	1 (6.2%)	
COPD				
Yes	9 (5.9%)	7 (5.1%)	2 (12.5%)	ns
No	132 (86.3%)	119 (86.9%)	13 (81.25%)	
Unknown	12 (7.8%)	11 (8.0%)	1 (6.25%)	
Cancer				
Yes	44 (28.8%)	38 (27.7%)	6 (37.5%)	ns
No	96 (62.8%)	87 (63.5%)	9 (56.25%)	
Unknown	13 (8.4%)	12 (8.8%)	1 (6.25%)	
Follow-up time (months)	43.8 ± 45.7	40.5 ± 43.4	84.7 ± 55.6	*p* = 0.005

*T2DM* type 2 diabetes mellitus, *IGT* Impaired Glucose Tolerance, *COPD* Chronic Obstructive Pulmonary Disease

Considering anthropometric data, most of patients were obese (BMI: 31.8 ± 6.1 kg/m^2^; 53.8%), with higher body weight in group B than in A (87.3 ± 18.2 vs 75.2 ± 16.8 kg; *p* < 0.03).

The prevalent order of classical comorbidities was arterial hypertension, dyslipidemia, dysglycemia (T2DM and IGT), osteoporosis, atrial fibrillation, with a frequency ranging 4.2–71.2%. We detected other three frequent associated diseases: other tumours (30.5%), COPD (5.1%), and glaucoma (2.8%).

No differences among subgroups were reported when considering the prevalence of comorbidities, except for higher prevalence in group B of glaucoma and dysglycemia (*p* < 0.01 for both) (Table 1). Comorbidities were not associated each other with the exception of glaucoma and dysglycemia that were closely to significance (*χ*^2^: 2.959, *p* = 0.079).

Notably, a non-neglectable number of patients (40 cases, 26.1%) were assuming statins for dyslipidemia.

### Hormonal characteristics

Hormonal characteristics of patients are reported in Table 2. The prevalence of ACS was 17.5% and the prevalence of primary aldosteronism was 4%.

**Table 2:** Hormonal characteristics of patients affected by AI (whole cohort and subgroups)

Variables	Total	Unchanged (A)	Changed (B)	*p* value
Urine free cortisol (mcg/24 h)	45.7 ± 46.5	45.8 ± 43.7	70.0 ± 73.8	ns
Urine cortisone (mcg/24 h)	92.1 ± 47.8	93.1 ± 47.5	71.7 ± 58.2	ns
Urine cortisol/urinary cortisone	0.32 ± 0.18	0.32 ± 0.19	0.34 ± 0.04	ns
Cortisol after DST (mcg/dl)	2.8 ± 2.8	2.9 ± 2.8	1.75 ± 0.07	ns
Direct Renin Concentration (DRC) (mcIU/ml)	20.6 ± 21.9	18.9 ± 20.2	42.5 ± 34.4	*p* < 0.04
Aldosterone (ng/dL)	18.2 ± 24.9	18.7 ± 25.6	11.1 ± 6.3	*p* < 0.001
Aldosterone Renin ratio (ARR)	6.1 ± 23.9	6.5 ± 24.8	0.44 ± 0.35	ns
Na (mEq/L)	140.8 ± 2.5	140.8 ± 2.5	140.5 ± 2.8	ns
K (mEq/L)	4.0 ± 0.4	4.0 ± 0.4	4.0 ± 0.25	ns
ACTH (pg/ml)	17.1 ± 13.0	17.1 ± 12.6	16.8 ± 18.0	ns
DHEAS (mcg/dl)	85.6 ± 97.4	83.0 ± 102.1	106.5 ± 44.5	*p* < 0.05
17 OH− progesterone (ng/ml)	1.4 ± 0.7	1.4 ± 0.8	1.4 ± 0.2	ns

*DST* 1 mg overnight dexamethasone suppression test

Cortisol secretion did not differ between the two groups, whereas in group B higher DHEAS (*p* < 0.05) and DRC (*p* < 0.04) levels were reported when compared to group A. On the contrary aldosterone was higher in group A than B (*p* < 0.001).

The prevalence of comorbidities was similar among groups, irrespective from detection of hormonal hypersecretion.

### Morphological characteristics

In 64.0% of cases the characterization of the incidental lesion was performed by CT scan, in 15.7% by MRI, in 17.0% with both methods (CT and MRI). Of them characterized by CT scan, mean density was 5.8 ± 20.1 HU. Accordingly, most of AIs had HU ≤ 10 (39.2%) while 20.9% had HU > 10 and 3.9% had mixed density as atypical adenoma.

At diagnosis, mean diameter was 32.2 ± 21.9 mm, larger in the group B than in group A (39.9 ± 21.5 vs 31.2 ± 21.9 mm, *p* < 0.05).

CT scan density was negatively correlated with aldosterone (*r* − 0.300, *p* = 0.05), sodium (*r* − 0.333, *p* = 0.003), and DHEAS levels (*r* − 0357, *p* < 0.05) in all the cohort.

### Surgical approach and histological diagnosis

Most of patients of group B underwent surgery respect to group A (87.5 vs 42.3%; *p* < 0.05). Two patients of group B refused surgery. Of these, the majority underwent a laparoscopic adrenalectomy (36.6%), followed by traditional laparotomic approach in 8 cases (5.2%), mainly in group B (*p* < 0.05). In 2 cases (1.3%) conversion from laparoscopic surgery to open approach was necessary due to intraoperative complications.

Histological diagnosis is reported in Table 3. A single case of adrenocortical carcinoma was found.

**Table 3:** Histological diagnosis of patients who underwent surgery in total number and percentage (whole cohort and subgroups)

Variables	Total *N* (%)	Unchanged (A) *N* (%)	Changed (B) *N* (%)	*p* value
Total	72 (100)	58 (80.5)	14 (19.5)	ns
Adenoma	48 (66.7)	39 (67.3)	9 (64.2)	ns
Adrenocortical carcinoma	1 (1.3)	0 (0)	1 (7.2)	ns
Adrenal hyperplasia	4 (5.5)	4 (7.0)	0 (0.0)	ns
Myolipoma	9 (12.5)	6 (10.4)	3 (21.4)	ns
Cyst	3 (4.7)	2 (3.4)	1 (7.2)	ns
Angiolipoma	1 (1.3)	1 (1.7)	0 (0.0)	ns
Ganglioneuroma	1 (1.3)	1 (1.7)	0 (0.0)	ns
Vascular malformation	2 (2.8)	2 (3.4)	0 (0.0)	ns
PEComa	1 (1.3)	1 (1.7)	0 (0.0)	ns
Adrenocortical oncocytoma	1 (1.3)	1 (1.7)	0 (0.0)	ns
Medullary hyperplasia	1 (1.3)	1 (1.7)	0 (0.0)	ns

## Phenotypic predictors

Analyzing the 16 cases of AIs with dimensional change over time (group B), cases were divided considering the time elapsed between diagnosis and enlargement: 31.3% of AIs changed at 6 months (*N* = 5); 25.0% changed at 12 months (*N* = 4); 12.5% changed at 24 months (*N* = 2); 31.3% changed at 36 months (*N* = 5). No subjects changed afterwards. No differences in the detection time of the dimensional increases of the lesions were shown within 36 months.

The presence of cortisol or aldosterone hypersecretion was not associated with tumour enlargement.

Using a Cox-regression model that included age, statin treatment, and one comorbidity, glaucoma (HR 13.34, CI 95% 3.19–55.70; *p* < 0.001), dysglycemia (HR 4.39, CI 95% 1.46–13.19; *p* < 0.008), and atrial fibrillation (HR 5.24, CI 95% 1.35–29.35; *p* < 0.01) were independent predictor of enlargement.

In a second model excluding statin treatment, and including presence of cortisol hypersecretion at the DST, all the variables (glaucoma: HR 8.85, CI 95% 1.30–58.70; *p* < 0.02; dysglycemia: HR 4.57, CI 95% 1.29–16.17; *p* < 0.01) remained significant with the exception of atrial fibrillation that was nearly to significance (HR 4.62, CI 95% 0.92–23.20; *p* = 0.062). A third model including primary aldesteronism instead cortisol hypersecretion did not modify significances revealed in the first model, also for atrial fibrillation.

**Fig. 1:**
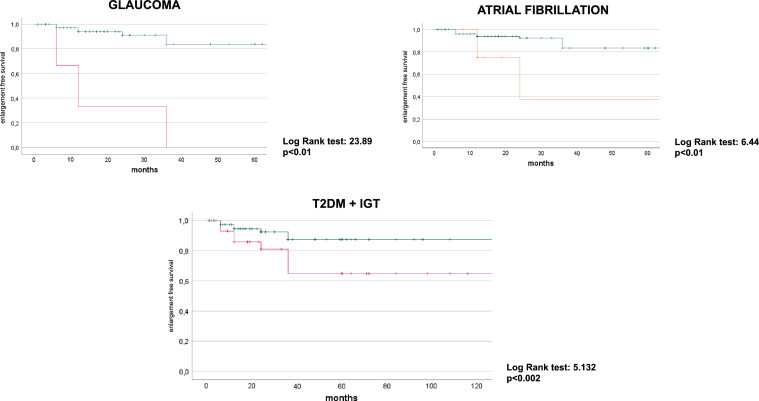
Dimensional change-free survival of patients affected by AI subdivided according to comorbidities (glaucoma, atrial fibrillation, dysglycemia)

Furthermore, subjects affected by glaucoma, atrial fibrillation, impaired glucose metabolism (T2DM or IGT) had a lower dimensional change-free survival than non-affected (Fig. 1).

## Discussion

The wide heterogeneity in clinical management of patients with AIs makes this condition challenging for clinicians [23]. Pitfalls still exist in recognizing both high-risk patients needing appropriate investigation and prompt discussion, and in streamlining the work-up for the low-risk patients who do not require further follow-up. Therefore, the aim of our study was to retrospectively collect radiological data and predictive factors of enlargement in a large cohort of patients.

First, we described a baseline and follow-up cohort of 153 patients. The majority of AIs did not change in size and the prevalence of significant AIs enlargement (> 5 mm) was 10.5% (16/153) in a mean follow-up of 35.2 months. Accordingly to literature, AIs enlargement was observed in up to 25% of patients (ranging from 7 to 26% and from 5 to 17% when considering an increase > 5 mm or > 10 mm, respectively) throughout a 2–5 years follow-up [24–26]. A recent meta-analysis by Elhassan et al. on more than 3000 subjects over a follow-up of 41.9 months described a minimal increase in 6.3% and a clinically significant increase (> 10 mm) in 2.5% of the patients, none developing adrenocortical carcinoma [20]. More recently, Ceccato et al. reported an increase ≥ 10 mm in 6/234 (2.5%) AIs in a median follow-up of 52 months [27]. The slight differences in prevalence of progression in studies described above should be explained by the different inclusion criteria (adrenal adenomas vs adrenal incidentaloma) and the different time of follow-up. Furthermore, our results confirm previous findings from longitudinal studies showing that the risk for developing adrenal carcinoma is very low, despite an increase in AI size [27].

When considering potential predictors of AI enlargement, patients that progressed had higher weight, DHEAS and DRC levels, while lower aldosterone level; the hormonal pattern is not surprising since tumours in patients affected by primary aldosteronism grow slowly. Furthermore, considering comorbidities, glaucoma and dysglycemia had higher prevalence in group B than A, and were also independent predictors of tumor enlargement.

Glaucoma is a well-known complication of exogenous and endogenous hypercortisolism, but no data are available either on non-functioning adrenal adenoma or in adenoma with ACS. The first description of association between Cushing syndrome and elevated intraocular pressure (IOP) was identified by Tartar in 1938 [28]. On the other way, Schwartz et al. noted that urinary free cortisol levels were higher in patients affected by open angle glaucoma than normal subjects [29]. Then, in 1974, Haas published the case of a 24-years-old patient who developed IOP, normalized after removal of a benign cortisol secreting adrenal adenoma [30]. The underlying pathophysiological mechanism might rely on glucocorticoids-induced alterations in the ocular trabeculae, thus impairing the aqueous humor reabsorption and increasing intraocular pressure [31]. Moving from this hypothesis, the presence of a subtle cortisol secretion (even with suppressed serum cortisol after 1 mg DST) in patients with AIs might contribute in explaining the onset of glaucoma in this clinical setting. Moreover, the possible association between glaucoma treatment (i.e., topical beta-blockers) and tumor enlargement should be an interesting research field. Considering other concomitant medications, a non-neglectable number of patients was assuming statins. Interestingly, HMG-CoA reductase inhibitors might play a protective role against dimensional changes for their anti-inflammatory and immunomodulatory role. Furthermore, the potential interfering role of statins in steroidogenesis been hypothesized in many studies. The study by Munkboel et al. conducted on mouse models has shown that atorvastatin is able to influence steroid homeostasis, resulting in a down-regulation of the StAR protein and the CYP11A1 enzyme, responsible for cleavage of cholesterol chain leading to pregnenolone [32]. A recent work by London et al. on 14 subjects, confirmed that multiple precursors of steroidogenesis decrease significantly 24 h after the first administration of pitavastatin, despite unsignificant plasma variation in cortisol and aldosterone levels [33]. This concern could be an intriguing topic of future research.

Considering dysglycemia, various evidences showed a causal role of insulin resistance and hyperinsulinemia on the growth of adrenal, even non-secreting, tumor. This hypothesis was first raised by Reincke et al. who demonstrated the presence of hyperinsulinism in patients with AIs [34]. Subsequently, it has been demonstrated that after an OGTT and hyperinsulinemic euglycemic clamp, patients with non-functioning adrenal adenomas were more insulin resistant than controls and that insulin sensitivity was inversely related to the tumor size [35], thus supporting the hypothesis that hyperinsulinemia may exert an anabolic effect on tumor growth. Furthermore, a correlation between peripheral insulin sensitivity and adrenal sensitivity to ACTH has been shown adding the idea that insulin resistance could inhibit adrenal response to ACTH, thus explaining the further increase in plasma ACTH levels and the resulting overstimulation of the adrenal cortex with adenoma growth [36]. On the other hand, the presence of a subtle cortisol secretion not detected with traditional methods could justify the role of dysglycemia on the risk of tumor enlargement. Moreover, persistent, chronic, low-grade inflammation leading to oxidative stress characterizing insulin resistance could be implicated in cell proliferation and tumor growth [37].

Apart subjects affected by glaucoma, and dysglycemia, also those with atrial fibrillation had a lower dimensional change-free survival than non-affected.

The role of atrial fibrillation (AF) has been recently reported and investigated. In particular, a recent study by Di Dalmazi et al. [38] pointed out a higher prevalence of atrial fibrillation in patients with ACS than in those with non-secreting adrenal masses and in patients with adrenal incidentalomas compared to the general population; during follow-up the risk of AF doubled in patients with ACS compared to patients with non-secreting adrenal adenomas. Thus, the authors concluded that AF should be considered as a part of the cardiovascular burden of patients with adrenal incidentalomas and ACS. In fact, recent researches have highlighted that exposure even to mild hypercortisolism is associated to a higher risk of developing cardiovascular diseases and related mortality, but the role of AF was not clearly demonstrated so far. The mechanisms that correlate the excess of cortisol with the onset of AF could be attributable to: (i) hyperactivation of mineralo- and glucocorticoid receptors [39]; (ii) direct effects on the cardiac excitation–contraction coupling by alteration of intracellular calcium levels by protein kinase C-dependent mechanisms [40]; (iii) effect on morphology and performance of cardiac muscle, including biventricular systolic dysfunction, altered left atrium ejection fraction, and dilated left atrium, which are predisposing conditions for development of AF [41].

The current study should be interpreted in the light of some limitations. The cohort does not allow deriving a causal relationship among variables. A larger sample size together with a longer follow-up could strengthen the result of the study. Moreover, we excluded patients with metastases and pheochromocytoma but the presence of lesions of different biological origin (i.e., adrenal hyperplasia, myolipoma, cyst, angiolipoma, vascular malformation) and subsequent high prevalence of patients undergoing surgery should be considered in the study limitations. Furthermore, considering the importance of the radiology, the recently proposed cutoff of 20 HU for unenhanced CT tumour attenuation should increase the accuracy of imaging characteristic assessment for exclusion of ACC [42]. Further prospective studies of validation are needed, especially in understanding the clinical impact of subtle undetectable cortisol hypersecretion on AIs enlargement and comorbidities. In fact, in the current study hormonal reassessment has not been considered, but morphological variations have been associated to functional changes over time in previous studies [24]. Moreover, the impact of other variables as concomitant medications (i.e., statins, antihypertensive treatments, topical beta-blockers) might have a role in tumor enlargement and should be analyzed in further retrospective and prospective studies.

In conclusion, our study pointed out that glaucoma might represent a new predictive factor for AI enlargement. This new evidence strengthens the importance of the phenotyping to personalize the diagnostic-therapeutic decisions. Furthermore, the multidisciplinary approach is the cornerstone of the management of these patients to ensure the best delivery of care.

## Data Availability

Available if requested.
